# Functional Near‐Infrared Spectroscopy Signal as a Potential Biomarker for White Matter Hyperintensity Progression in Patients With Subcortical Vascular Cognitive Impairment: A Pilot Study

**DOI:** 10.1002/brb3.70598

**Published:** 2025-05-30

**Authors:** Qi Wang, Byoung‐Soo Shin, Sun‐Young Oh, Seungbae Hwang, Ko Woon Kim

**Affiliations:** ^1^ Department of Medicine, Medical School Jeonbuk National University Jeonju Republic of Korea; ^2^ Department of Neurology Jeonbuk National University Medical School and Hospital Jeonju Republic of Korea; ^3^ Research Institute of Clinical Medicine of Jeonbuk National University‐Biomedical Research Institute of Jeonbuk National University Hospital Jeonju Republic of Korea; ^4^ Department of Radiology Jeonbuk National University Medical School and Hospital Jeonju Republic of Korea

**Keywords:** cognitive test, mild cognitive impairments, near‐infrared spectroscopy, small vessel disease

## Abstract

**Background:**

White matter hyperintensities (WMH) are a common cause of subcortical vascular cognitive impairment (SVCI). The silent yet progressive nature of WMH in cognitive decline underscores the need for reliable biomarkers for early detection and monitoring of its progression. This study aims to investigate the association between functional near‐infrared spectroscopy (fNIRS) signals during mental and physical activities and WMH volume. Additionally, it explores the relationship between fNIRS signals and WMH progression.

**Material and Methods:**

We recruited 27 patients with mild cognitive impairment (MCI) presenting WMH clinical characteristics. Data from fNIRS and MRI scans were collected during their first visit. Ten of them underwent fNIRS and MRI scans in a second visit two years later. WMH volume analysis used volBrain lesionBrain 1.0 (https://www.volbrain.net). ROC curve analysis was applied to the normalized WMH volume to determine a cut‐off value for distinguishing between the subcortical vascular MCI (svMCI) and amnestic MCI (aMCI) groups. We compared fNIRS data during cognitive tests and physical activities between svMCI and aMCI groups at the first visit and in the two‐year follow‐up.

**Results:**

While cognitive profiles were similar between groups, svMCI patients showed significantly reduced fNIRS signals, particularly in the left orbitofrontal cortex (OFC) during verbal fluency tasks (*P* = 0.005), with further reductions in the left dorsolateral prefrontal cortex (*P* = 0.049), left OFC (*P* = 0.012), and right OFC (*P* = 0.02) over two years. Baseline WMH volume correlated negatively with fNIRS signals during the Stroop test (*r* = ‐0.837, *P* = 0.005). Changes in WMH volume over two years correlated positively with changes in fNIRS signals in the right ventrolateral prefrontal cortex during memory tasks (*r* = 0.886, *P* = 0.033) and left OFC during balance tasks (*r* = 0.786, *P* = 0.028).

**Conclusion:**

Our results suggest that fNIRS signals have the potential to serve as biomarkers for WMH progression.

## Graphical abstract

This study explores the relationship between fNIRS signals and WMH volume, investigating their potential as biomarkers for WMH progression. It highlights differential prefrontal activation patterns in amnestic and subcortical vascular MCI groups and demonstrates a positive correlation between WMH changes and fNIRS signals over two years during cognitive and physical tasks.

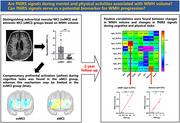

## 1 Introduction

Cerebral small vessel disease (CSVD) refers to pathological processes affecting the structural and functional integrity of small arteries, arterioles, venules, and capillaries within the brain (Duering et al. 2023), which contribute significantly to the subcortical vascular cognitive impairment (SVCI) (Hamilton et al. 2021). White matter hyperintensity (WMH) is considered the most common and significant neuroimaging feature of SVCI (Wardlaw et al. 2013; Wardlaw et al. 2015). However, WMH is the result of disease progression, and there is a lack of predictive tools for disease progression and responses to therapy. Therefore, it is necessary to develop reliable biomarkers for the early detection of SVCI, monitor disease progression, especially WMH progression, and eventually contribute to the development of appropriate treatments and interventions.

SVCI refers to a spectrum of cognitive impairment ranging from the intermediate stage of mild cognitive impairment (MCI) to subcortical vascular dementia (SVaD) (Skrobot et al. 2018). Therefore, subcortical vascular MCI (svMCI), the intermediate stage before SVaD, is as important as amnestic MCI (aMCI), the intermediate stage before Alzheimer's disease, but its pathophysiology and clinical progression are different from those of aMCI. Neuroimaging shows that WMH is typically more extensive and severe in svMCI than in aMCI (Acharya et al. 2019). Cross‐sectional studies found that a more severe WMH burden is associated with lower CBF (Shi et al. 2016). However, it remains debated whether CBF is associated with WMH progression (Stewart et al. 2021). The differences in study results could be due to variations in imaging modalities and WMH or CBF measurement protocols.

The association between CBF and WMH using various imaging modalities has been demonstrated in patients with WMH. For example, an arterial spin labeling (ASL) magnetic resonance imaging (MRI) study revealed a strong association between CBF and WMH volume in patients with MCI (Kim et al. 2020). A single‐photon emission computed tomography (SPECT) study showed that Alzheimer's disease (AD) patients with WMH exhibited significantly decreased CBF in the anterior cingulate gyrus and insula compared to AD patients without WMH (Kimura et al. 2012). Additionally, a transcranial Doppler ultrasonography (TCD) study demonstrated reduced mean blood flow velocity, widely used as a proxy for CBF, in patients with vascular depression and severe WMH (Puglisi et al. 2018). Similarly, a positron emission tomography (PET) study found that the severe WMH group exhibited lower CBF in the centrum semiovale compared with the mild WMH group (Nezu et al. 2012). These methodologies have been well established for examining the relationship between CBF and WMH during the resting state, but they are limited when applied to task performance, particularly due to motion artifacts.

The correlation between WMH and reactive CBF during tasks could be explored using functional near‐infrared spectroscopy (fNIRS), a non‐invasive neuroimaging technique that uses near‐infrared light to assess brain oxygenation by leveraging the distinct absorption spectra of molecules within the brain. Cerebral hemodynamic parameters, including oxy‐hemoglobin (HbO) and deoxy‐hemoglobin (HbR) measured by fNIRS, have been observed to be highly correlated with CBF (Agbangla et al. 2017; Villringer et al. 1993; Wyatt et al. 1990). Thus, fNIRS parameters are associated with WMH. There are several practical benefits to fNIRS over more widely recognized techniques such as ASL‐MRI and PET, including tolerance to motion artifacts, portability, and better temporal resolution (Pennekamp et al. 2009; Wintermark et al. 2005). These advantages have facilitated its application in studying cognitive impairment, with recent studies demonstrating its utility in distinguishing aMCI from healthy controls through multimodal analysis (Cheng et al. 2024) and identifying altered prefrontal activation patterns in Parkinson's disease‐related MCI (Shu et al. 2024). Additionally, its capability to capture neurovascular responses during tasks, owing to its high temporal resolution, renders it sensitive for detecting alterations in brain perfusion associated with CSVD (Moretti and Caruso 2020).

This study aimed to explore whether fNIRS signals during mental and physical activities were associated with WMH volume. Additionally, this study investigated the relationship between the fNIRS signal and WMH progression and further investigated the fNIRS signal as a potential biomarker of WMH progression.

## 2 Methods

### 2.1 Participant Recruitment

In this study, 27 patients with MCI who presented with WMH were recruited from the Memory Disorder Clinic at Jeonbuk National University Hospital between February 4^th^, 2021, and February 28^th^, 2023. As this is a pilot study to explore the association of fNIRS signals and the progression of WMH, the sample size was determined according to recommendations for planning a pilot study (Cocks and Torgerson 2013; Whitehead et al. 2016). Patients with MCI met the criteria proposed by Peterson and colleagues (Petersen 2004) with the following modifications: The inclusion criteria for MCI patients in this study were as follows: (1) a subjective cognitive complaint by the patient or caregiver, (2) an objective cognitive impairment below the 16^th^ percentile of education‐ and age‐matched norms in at least one of 4 domains (memory, visuospatial, language, or frontal‐executive function) of the Seoul Neuropsychological Screening Battery (SNSB) (Kang et al. 2019), (3) normal activities of daily living (ADL) determined clinically and according to the instrumental ADL scale (Kang et al. 2002), and (4) no dementia. All patients underwent clinical interviews, neurological examinations, neuropsychological batteries, brain MRI, and fNIRS at baseline. Patients were also evaluated using laboratory tests, including complete blood count, blood chemistry, thyroid function tests, vitamin B_12_, folate, and syphilis serology. The exclusion criteria in this study were as follows: (1) patients with structural lesions such as territorial infarction, intracranial hemorrhage, brain tumor, and hydrocephalus observed on brain MRI and (2) patients with any abnormal results of the previously described laboratory tests. Based on baseline WMH volume, patients were classified into subcortical vascular MCI (svMCI) and amnestic MCI (aMCI) groups. Ten of the 27 patients underwent follow‐up brain MRI and fNIRS after 2 years. This study was approved by the Institutional Review Board of the Jeonbuk National University Hospital (2020‐12‐023). All the participants provided written informed consent after the study objectives were explained.

### 2.2 MRI

At both baseline and the 2‐year follow‐up, all participants underwent scanning using a 3.0‐Tesla scanner (Magnetom Verio, Siemens Healthcare, Erlangen, Germany) with a 12‐channel head coil at Jeonbuk National University Hospital. The acquisition parameters of the three‐dimensional T1 Turbo Field Echo image were as follows: repetition time (TR) = 8.6 ms, echo time (TE) = 4.6 ms, flip angle (FA) of 8°, the field of view (FOV) = 256×256 mm, slice thickness = 1.0 mm. The acquisition protocol for the T2 fluid‐attenuated inversion recovery (FLAIR) image was as follows: TR = 11000 ms, TE = 125 ms, FA = 90°, FOV = 230×230 mm, and slice thickness = 2.0 mm.

### 2.3 White Matter Hyperintensity Volume Analysis

**FIGURE 1:**
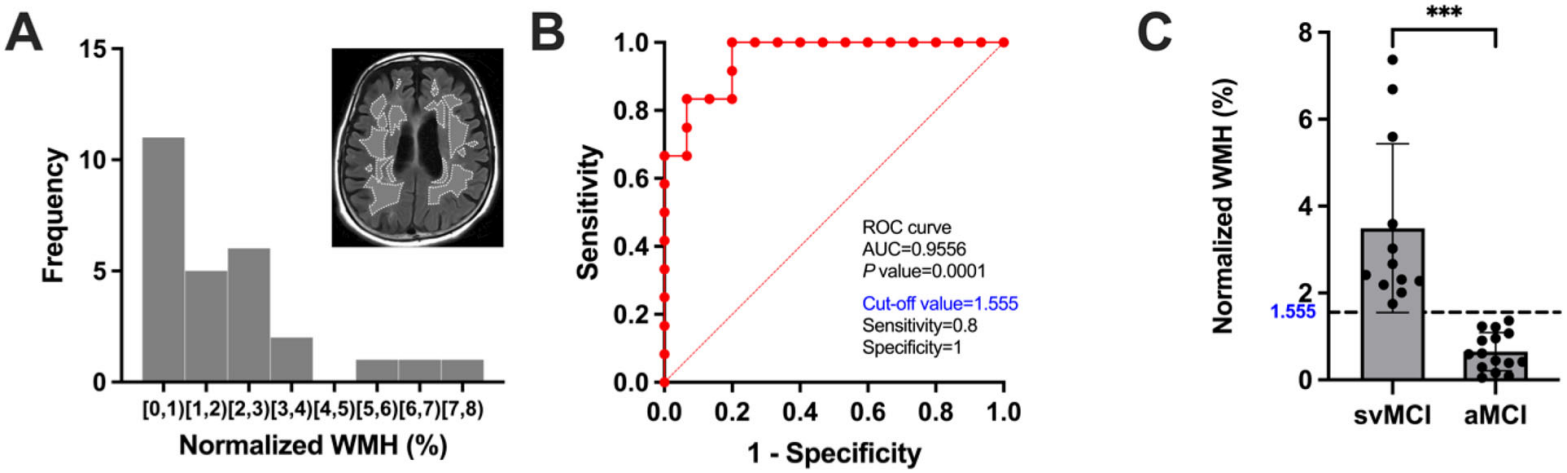
**Identifying subcortical vascular mild cognitive impairment (svMCI) from amnestic mild cognitive impairment (aMCI)**. (A) The distribution of normalized white matter hyperintensity (WMH) volume at baseline. (B) The receiver operating characteristic (ROC) curve showed the optimal cut‐off value of WMH to svMCI from aMCI (1.555%). (C) Finally, we identified 15 svMCI patients by selecting patients who had a normalized WMH value greater than 1.555%. The bar graph shows that the normalized WMH of svMCI patients is significantly higher than that of aMCI patients.

WMH was evaluated both manually and automatically. Manually, WMH on FLAIR images was defined using the modified Fazekas ischemia criteria (Noh et al. 2014; Pantoni et al. 2005). Automatically, an analysis of WMH volume was conducted using the volBrain lesionBrain 1.0 version (https://www.volbrain.net), a fully automated segmentation software specifically designed for segmenting white matter lesions from MRI data (T1‐weighted and T2 FLAIR images); its precision and stability have been evaluated in the MSSEG MICCAI Challenge 2016 (Commowick et al. 2018; Coupé et al. 2018). Subsequently, the WMH volumes were normalized by dividing them by the total intracranial volume. Based on the results acquired from volBrain, it was evident that the distribution of WMH volume in most patients was concentrated within the range of 0–4% (Figure 1A). Receiver Operating Characteristic (ROC) curve analysis determined a cutoff value of 1.555% (normalized WMH volume) for classifying patients into the svMCI and aMCI groups. The ROC curve displayed excellent discriminatory performance, boasting a high area under the curve (AUC) value of 0.9556 (Figure 1B). Significant differences were observed in the comparison of normalized WMH volumes between the svMCI group and the aMCI group (*P* < 0.001; Figure 1C).

### 2.4 Neuropsychological Assessments

The SNSB consists of tests for attention, language, visuospatial function, verbal and visuospatial memory, frontal‐executive function, and general cognition, such as the Mini‐Mental State Examination (MMSE), Clinical Dementia Rating (CDR), and CDR sum of boxes. Attention was assessed with the forward and backward digit span tests; language was assessed with the Korean version of the Boston Naming Test (K‐BNT) and calculations; visuospatial function was assessed by copying the Rey‐Osterrieth Complex Figure Test (RCFT); verbal and visuospatial memory function was assessed with immediate recall, delayed recall, recognition of the Seoul Verbal Learning Test (SVLT), and RCFT; and frontal‐executive function was assessed using the Controlled Oral Word Association Test (COWAT) and Stroop color reading test. Each score was converted to a standardized Z‐score based on age‐ and education‐adjusted norms.

### 2.5 Experimental Design

We collected fNIRS signal data during the cognitive and physical activities detailed below. During the testing procedure, measurements were taken to ensure consistent placement of the device on the participant's forehead, targeting the same brain region. To achieve this, a reference point located in front of the device was aligned with the center of the participant's eye. Additionally, to minimize potential interference from ambient light sources such as lamplight or sunlight, participants were instructed to perform cognitive and physical tasks in a dimly lit room. To minimize the impact of hair on the forehead area, the participants’ hair was gently combed away from the forehead before placing the device. Once the device was properly positioned, the strap and Velcro hooks on the back were securely fastened to ensure stability and prevent movement during testing.

For the cognitive tests, the participants were instructed to sit on a chair and maintain a comfortable posture in a quiet and dimly lit environment. Participants completed four cognitive tasks using a tablet computer connected to an fNIRS device via wireless transmission. Before starting the task, the participants focused on a cross pattern displayed on the tablet screen for 3 min to collect resting‐state data. The four cognitive tasks are the same as those used in our previous studies, and their validity has already been verified (Kim et al. 2018; Wang et al. 2023):(1) verbal fluency test (VFT); (2) digit span backward task (DST); (3) Korean‐color word Stroop Test (K‐CWST), including congruent (STRC) and incongruent (STRI) conditions; and (4) Social Event Memory Test (SEMT), as depicted in Supplementary Information S1 and Supplementary Table 1. A 30 s rest period was provided between each task.

In terms of the physical activities, participants were required to perform a “Balance Task” followed by a “Squat Task.” In the “Balance Task,” we measured changes in fNIRS signals while participants opened their arms and balanced on one leg for 20 s with eyes open and for 20 s with eyes closed. In the “Squat Task,” participants were measured for changes during 60 s of standing and 60 s of squatting.

### 2.6 Experimental Apparatus

This study utilized a noninvasive, wearable, head‐mounted fNIRS system called NIRSIT, developed by OBELAB Inc. (Seoul, Korea) (Shin et al. 2017). The system enables the acquisition of oxyhemoglobin (HbO_2_) values from the prefrontal region of the human brain. The fNIRS system employed in this study consisted of 24 laser sources emitting at wavelengths of 780/850 nm, with a maximum power below 1 mW, and 32 photodetectors with variable source‐detector spacing (1.5, 2.12, 3.0, and 3.35 cm). The fNIRS system generated 204 measurement points and captured data at a sampling rate of 8.138 Hz. The oxygen saturation levels in the prefrontal cortex blood, ranging from 15% to 95% within 5 s of updated time, were wirelessly transmitted and stored in the connected tablet device during both the resting and task periods.

### 2.7 fNIRS Signals Data Analysis

NIRSIT Analysis Toolbox v3.6.2. (OBELAB Inc.) was used to analyze the fNIRS signals. Given that previous research has established that an optimal depth of NIR light penetration into the cortex can be achieved using a source‐detector distance of approximately 3 cm, and considering that this channel configuration is widely employed in fNIRS studies investigating cognitive tasks (Ferrari and Quaresima 2012), a total of 48 channels were established in this study, with a separation distance of 3.0 cm between the sources and detectors.

HbO_2_ saturation was assessed in each channel using the Modified Beer‐Lambert law (Baker et al. 2014; Cope et al. 1988). To minimize confounding factors such as cardiovascular artifacts and environmental noise, the data were filtered with high‐pass and low‐pass filters set at 0.005 Hz and 0.1 Hz, respectively. Baseline correction was performed using the last 5 s of the pre‐task period as the baseline reference for each block. Before extracting the hemodynamic data, channels with poor signal quality, indicated by a signal‐to‐noise ratio of less than 30 dB, were excluded to ensure accurate interpretation.

Both intra‐ and inter‐individual analyses of HbO_2_ values were conducted using both the 1^st^‐level general linear model (GLM) and the 2^nd^‐level GLM. Specifically, in the 1^st^‐level GLM, a design matrix X was constructed by incorporating task‐related regressors of interest as well as nuisance regressors such as motion‐related parameters, time‐dependent drift, and run‐wise errors. The GLM was then fitted to extract the fNIRS signal of interest and eliminate the confounding factors. In the 2^nd^‐level GLM, contrast was defined based on the task time blocks, and the linear combination of the contrasted fNIRS signal associated with event regressors was determined using time‐series HbO_2_ data for each subject. This fNIRS signal, which signifies the neural activity in experimental conditions, was used for further statistical analysis to examine the differences between the svMCI and aMCI groups.

The fNIRS device featured 48 channels, which were subdivided into eight distinct subregions: the left and right dorsolateral prefrontal cortex (DLPFC), ventrolateral prefrontal cortex (VLPFC), frontopolar prefrontal cortex (FPC), and orbitofrontal cortex (OFC), as illustrated in Supplementary Figure 1. Subsequently, the mean values of the fNIRS data for each of the eight Brodmann regions were calculated for further statistical analyses based on the respective channels.

### 2.8 Statistical Analysis

Normality and variance heterogeneity were tested for demographic and clinical characteristics. The Mann‐Whitney U‐test was applied to non‐parametric continuous variables, while Fisher's exact test was used for categorical variables. Regarding cognitive performance data, independent 2‐sample t‐tests were used for parametric variables, and two‐sample Wilcoxon tests were employed for nonparametric variables. ROC curves were employed to determine the optimal cutoff value for distinguishing between the svMCI and aMCI groups based on WMH volume. Subsequently, the Mann‐Whitney U‐test was used to compare the WMH volume between the two groups.

For the fNIRS data, an independent 2‐sample t‐test was used to compare the svMCI and aMCI groups. However, for Brodmann regions where normality was not satisfied (assessed using the Shapiro‐Wilk test), we applied the Mann‐Whitney U‐test instead. To account for multiple comparisons, we applied false discovery rate (FDR) correction using the Benjamini‐Hochberg procedure (Benjamini and Hochberg 1995), which controls the expected proportion of false positives among the detected significant channels, making it a more powerful method for multiple comparison correction in fNIRS analysis. Spearman's rank correlation analysis was used to assess the relationship between the fNIRS data and WMH volume.

All statistical analyses were performed using SPSS version 27 (IBM Corp., Armonk, NY, USA) and GraphPad Prism 9.5.1 (GraphPad Software, San Diego, CA, USA). A p‐value < 0.05 was considered significant.

## 3 Results

### 3.1 Demographics and Clinical Characteristics

This study included a total of 27 patients diagnosed with MCI who presented with WMH. All participants were Asian. Participants were categorized into the svMCI group (n = 12) and the aMCI group (n = 15) based on baseline WMH volume. Regarding the manually calculated WMH volume, there were three severe and nine moderate participants in the svMCI group, and one moderate and 14 mild participants in the aMCI group using a modified Fazekas scale. In terms of automatically calculated WMH volume, median WMH volume by total intracranial volume (normalized WMH volume) was 2.55 % (2.21, 5.10) in the svMCI group and 0.59 % (0.29, 1.07) in the aMCI group, using the volBrain lesionBrain 1.0 version (https://www.volbrain.net). There were no significant differences between the svMCI and aMCI groups in terms of age, sex, years of education, APOE4 carrier, hypertension, diabetes mellitus, or cognitive profiles, including, MMSE scores, Clinical Dementia Rating Scale (CDR), CDR sum of box, attention, language, visuospatial function, memory, and frontal/executive functions. Demographic information and clinical characteristics of the participants are presented in Table 1.

**TABLE 1:** Demographics and clinical characteristics.

	svMCI (N =12)	aMCI (N =15)	*P‐*values
**Age (IQR)**	76 (74.5, 78.75)	71 (63.5, 75.5)	0.492^a^
**Sex (F:M)**	11:1	13:2	>0.999^c^
**Education (IQR)**	6 (0, 7.5)	9.5 (6, 15)	0.538
**APOE4 carrier**	5/6 (83.3%)	6/6 (100%)	>0.999
**Hypertension**	11	10	0.182^c^
**Diabetes mellitus**	4	1	0.139^c^
**WMH volume**			
*baseline*			
Manually calculated (modified Fazekas, mild: moderate: severe)	0: 9: 3	14: 1: 0	
Automatically calculated (%, normalized)	2.55 (2.21, 5.10)	0.59 (0.29, 1.07)	<0.001
*2‐year follow‐up*			
Manually calculated (modified Fazekas, mild: moderate: severe)	0: 2: 2	4: 2: 0	
Automatically calculated (%, normalized)	3.76 (4.59, 3.44)	0.92 (1.51, 0.17)	0.002
**Cognitive profiles**			
MMSE (IQR)	26 (23.75, 27)	26.5 (24, 28.75)	0.486^a^
CDR (IQR)	0.5 (0.5, 0.5)	0.5 (0.5, 0.5)	>0.999
CDR sum of box (IQR)	1.5 (0.5, 1.5)	1.5 (0.625, 1.875)	0.057
*Attention*			
Forward digit span (IQR)	4 (4, 6)	4.5 (4, 6.75)	0.316
Backward digit span (IQR)	3 (2, 3.25)	3 (3, 4)	0.103
*Language*			
K‐BNT (IQR)	32.5 (29.75, 43.25)	44 (36, 50)	0.588^a^
Calculation (IQR)	8 (5.5, 11.25)	10.5 (8.5, 12)	0.771
*Visuospatial function*			
RCFT: copying (IQR)	24.75 (20.75, 33.25)	31 (24.75, 34.75)	0.229^b^
*Memory*			
SVLT: immediate recall (IQR)	15.5 (13.5, 18.25)	15 (13, 25)	0.261
SVLT: delayed recall (IQR)	3 (1.75, 5.25)	2.5 (0, 5.75)	0.098
SVLT: recognition (IQR)	18 (15.75, 20.25)	20.5 (18, 22)	0.935^b^
RCFT: immediate recall (IQR)	5.75 (1.125, 10.5)	8.75 (2.5, 11.875)	0.309
RCFT: delayed recall (IQR)	2.75 (0, 7.875)	9 (2.625, 12)	0.181
RCFT: recognition (IQR)	18 (16, 19)	19 (17, 20.5)	0.745^b^
*Frontal/executive functions*			
COWAT animal (IQR)	11.5 (9.5, 15.5)	13 (11.25, 17)	0.583^a^
COWAT supermarket (IQR)	14 (12.75, 15.25)	16 (12.5, 21)	0.814
COWAT phonemic (IQR)	13 (8.5, 17)	10.5 (5.25, 23)	0.622
Stroop test: color (IQR)	60 (46, 74.5)	65.5 (55.5, 103.75)	0.467^b^

*Notes*: Data are presented as the median and interquartile range (IQR) for continuous variables. The Mann‐Whitney U‐test was used for comparisons.

Abbreviations: svMCI, subcortical vascular mild cognitive impairment; aMCI, amnestic mild cognitive impairment; K‐BNT, Korean version of the Boston naming test; RCFT, Rey‐Osterrieth Complex Figure Test; SVLT, Seoul Verbal Learning Test; COWAT, Controlled Oral Word Association Test; MMSE, Mini‐Mental State Examination; CDR, Clinical Dementia Rating Scale; WMH, White Matter Hyperintensities; CREDOS: Clinical Research Center for Dementia of South Korea.

^a^ independent samples t‐test.

^b^ Satterthwaite t‐test.

^c^ Fisher exact test.

A significant difference in WMH volume (*P*<0.001) was found between the svMCI and aMCI groups at baseline. There were no significant differences in age, sex, years of education, or neuropsychological assessment results.

APOE was analyzed in 12 participants: six with svMCI and six with aMCI. Participants with one or more copies of the ε4 allele (i.e., ε2/4, ε3/4, ε4/4) were ε4 carriers.

### 3.2 Comparison of Cognitive Performance

Participants performed four cognitive tasks (VFT, DST, K‐CWST, and SEMT). The number of responses during the VFT task was recorded. For the other tasks, accuracy and response time were collected (Table 2). At baseline, the svMCI group exhibited lower accuracy in the STRC task (*P* = 0.038) and longer response times in the SEMT task than the aMCI group (*P* = 0.019). At the 2‐year follow‐up, the svMCI group displayed lower accuracy than the aMCI group in the STRI task (*P* = 0.045). No significant differences were observed between the svMCI and aMCI groups in DST and VFT scores during either visit.

**TABLE 2:** Comparison of cognitive performance.

Task	Baseline (mean ± SD)			2‐year follow‐up (mean ± SD)		
	svMCI	aMCI	*P*‐value	svMCI	aMCI	*P*‐value
**VFT**						
Number of responses	6.92±4.96	10.47±6.96	0.178	6.75±4.03	19.33±13.13	0.068
**DST**						
Accuracy	0.26±0.24	0.40±0.36	0.21	0.45±0.19	0.64±0.36	0.316
Response time (ms)	8372.85±2419.56	7327.75±1978.75	0.24	7365.96±2248.03	6386.76±1872.95	0.501
**STRC**						
Accuracy	0.97±0.02	0.98±0.02	0.038	1.00±0.00	0.98±0.02	0.172
Response time (ms)	1722.11±221.92	1673.79±496.18	0.746	1536.55±442.87	1337.34±267.13	0.467
**STRI**						
Accuracy	0.68±0.31	0.80±0.25	0.147	0.75±0.14	0.98±0.02	0.045
Response time (msec)	2654.10±930.66	2719.89±1545.84	0.614	2829.67±657.79	2052.93±1085.59	0.197
**SEMT**						
Accuracy	0.43±0.23	0.57±0.25	0.139	0.32±0.20	0.62±0.37	0.135
Response time (ms)	15106.21±3612.95	11296.00±4024.79	0.019	13364.93±1506.44	9242.19±5223.00	0.121

Abbreviations: aMCI, amnestic mild cognitive impairment; DST, Digit span backward test; SD, standard deviation; SEMT, social event memory test; STRC: Korean‐Color Word Stroop Test—Congruent; STRI: Korean‐Color Word Stroop Test—Incongruent; svMCI, subcortical vascular mild cognitive impairment; VFT, Verbal Fluency test.

### 3.3 Group Comparisons of fNIRS Signals Data

**FIGURE 2:**
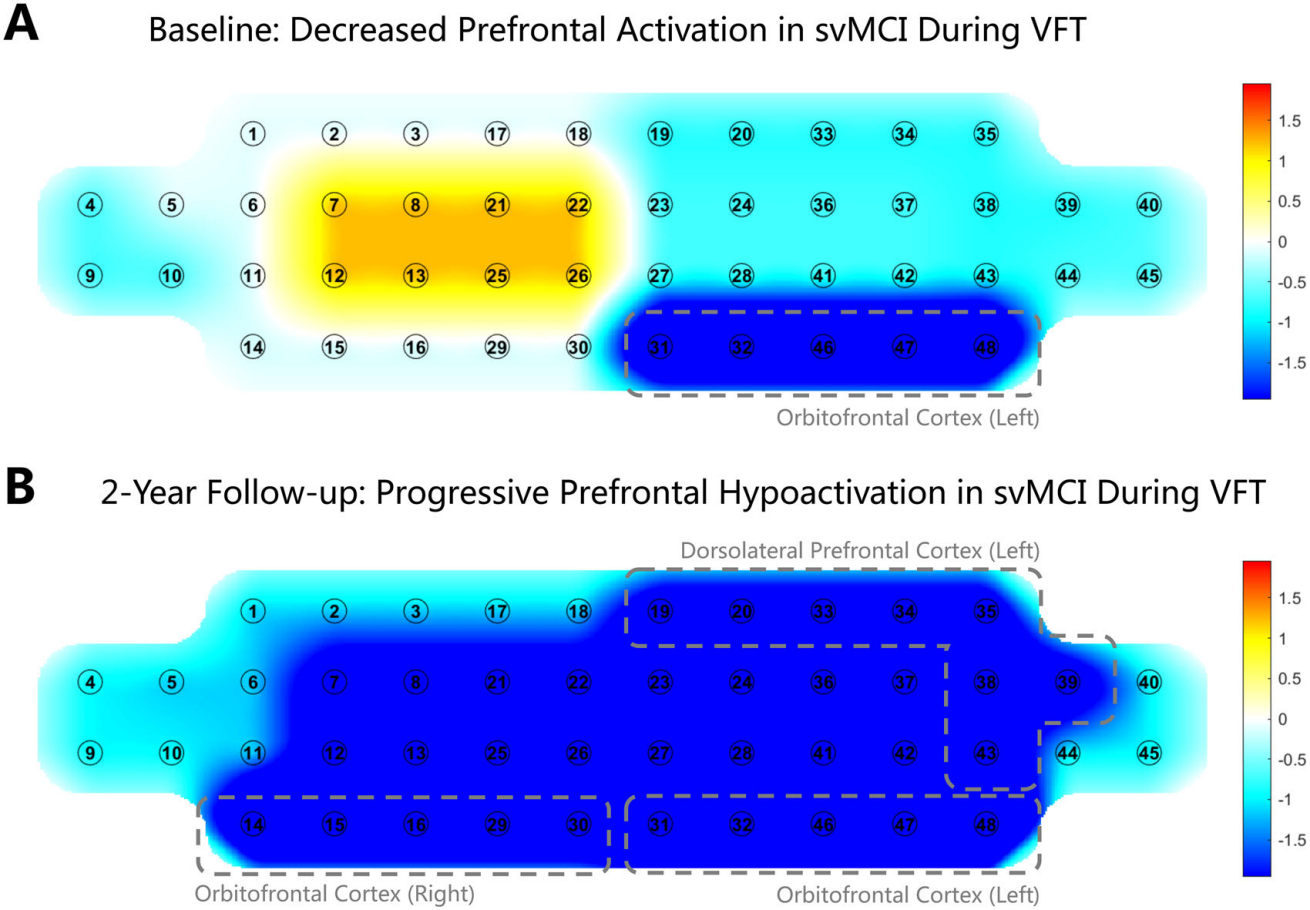
**Comparison of functional near‐infrared spectroscopy (fNIRS) data during the Verbal Fluency Test (VFT) between subcortical vascular mild cognitive impairment (svMCI) and amnestic mild cognitive impairment (aMCI) at baseline and 2‐year follow‐up**. The red color indicates increased fNIRS data, and the blue color indicates decreased fNIRS data in the svMCI group. (A) At the baseline, fNIRS data of svMCI patients was significantly decreased compared to that of aMCI patients in the left orbitofrontal cortex (OFC) (T = ‐3.989, Cohen's d = ‐1.74, 95% CI [‐0.74, ‐2.75], *P* = 0.005). (B) At the 2‐year follow‐up, fNIRS data of svMCI patients was significantly decreased compared to that of aMCI patients in the bilateral prefrontal cortex, specifically in the left dorsolateral prefrontal cortex (DLPFC) (T = ‐2.965, Cohen's d = ‐2.11, 95% CI [‐0.37, ‐3.85], *P* = 0.049), left OFC (T = ‐5.746, Cohen's d = ‐4.85, 95% CI [‐1.57, ‐8.13], *P* = 0.012), right OFC (T = ‐4.071, Cohen's d = ‐3.10, 95% CI [‐0.88, ‐5.33], *P* = 0.02).

HbO_2_ changes during cognitive and physical tasks were assessed using fNIRS at baseline and at the 2‐year follow‐up. At baseline, during the VFT task, the svMCI group exhibited a significantly decreased fNIRS signal compared to the aMCI group in the left OFC (T = ‐3.989, Cohen's d = ‐1.74, 95% CI [‐0.74, ‐2.75], *P* = 0.005; Figure 2A). During the SEMT task, the svMCI group exhibited a significantly increased fNIRS signal compared to the aMCI group in the left VLPFC (T = 3.526, Cohen's d = 1.962, 95% CI [3.290, 0.635], *P* = 0.03; Supplementary Figure 2B‐1). No significant differences were observed between the svMCI and aMCI groups in the DST, Stroop, and two physical tasks (Supplementary Figure 2).

At the 2‐year follow‐up, during the VFT task, the svMCI group exhibited a significantly decreased fNIRS signal compared to the aMCI group in the bilateral prefrontal cortex (left DLPFC, T = ‐2.965, Cohen's d = ‐2.11, 95% CI [‐0.37, ‐3.85], *P* = 0.049; left OFC, T = ‐5.746, Cohen's d = ‐4.85, 95% CI [‐1.57, ‐8.13], *P* = 0.012; right OFC, T = ‐4.071, Cohen's d = ‐3.10, 95% CI [‐0.88, ‐5.33], *P* = 0.02. Figure 2B). During the balance task, the svMCI group exhibited a significantly increased fNIRS signal in the right OFC (T = 5.820, Cohen's d = 5.432, 95% CI [9.394, 1.471], *P* = 0.022; Supplementary Figure 2E‐2). No significant differences were observed between the svMCI and aMCI groups in the DST, Stroop, SEMT, and Squat tasks (Supplementary Figure 2).

### 3.4 Correlation Between WMH Volume and fNIRS Signals

**FIGURE 3:**
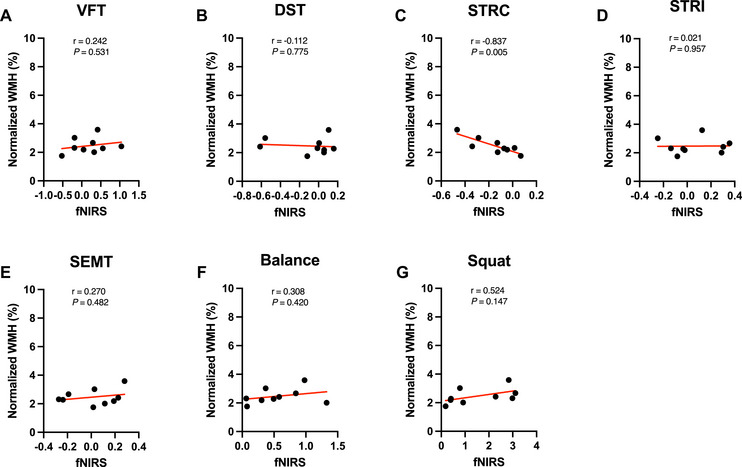
**The correlation between baseline functional near‐infrared spectroscopy (fNIRS) data and white matter hyperintensity (WMH) volume during cognitive tasks and physical tasks for the svMCI group**. (A) Verbal Fluency Test (VFT), (B) Digit span backward task (DST), (C) Korean‐Color Word Stroop Test Congruent Condition (STRC) and (D) incongruent condition (STRI), (E) Social Event Memory Test (SEMT), (F) balance task, and (G) squat task. Only in the Korean‐Color Word Stroop Test Congruent Condition (STRC), there was a strongly negative correlation between WMH volume and the fNIRS data (r = ‐0.837, 95% CI [‐0.965, ‐0.389], *P* = 0.005).

We assessed the relationship between baseline WMH volume and fNIRS signals in the svMCI group using Spearman's rank correlation. A strong negative correlation was detected between baseline WMH volume and fNIRS signals during the STRC task (r = ‐0.837, 95% CI [‐0.965, ‐0.389], *P* = 0.005) in the svMCI group; however, no significant correlation was found in the other tasks, as shown in Figure 3. No significant correlations were observed in the aMCI group. Three outliers were excluded due to severe WMH burden (modified Fazekas grade 3), as previous studies have shown that severe WMH burden may cause alterations in CBF distinct from those observed in mild‐to‐moderate WMH cases (Huang et al. 2021). The correlation between baseline WMH volume and fNIRS signals during the STRC task was not significant when outliers were included (Supplementary Figure 3C).

**FIGURE 4:**
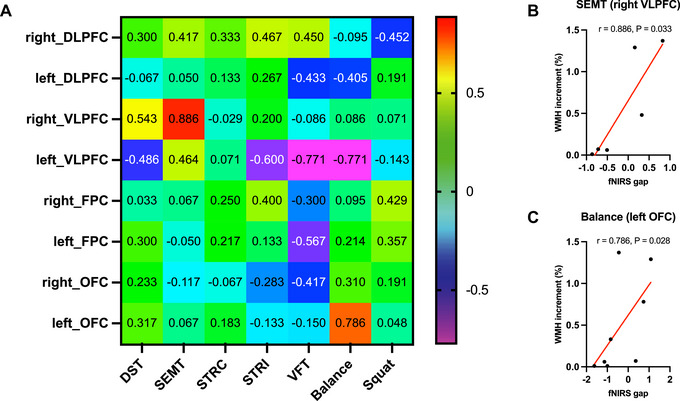
**The correlation between white matter hyperintensities (WMH) volume increment and functional near‐infrared spectroscopy (fNIRS) data gap of the baseline and 2‐year follow‐up during cognitive and physical tasks**. (A) The heat map visualizes Spearman correlation results between WMH volume increment and fNIRS data gap. Warmer colors represent positive correlations, while cooler colors indicate negative correlations. Each cell in the map represents the correlational coefficient in a specific Brodmann area during a specific cognitive or physical task. (B) In the Social Event Memory Task (SEMT), WMH volume increment had a strong positive correlation with the fNIRS data gap in the right ventrolateral prefrontal cortex (VLPFC, r = 0.886, 95% CI [0.264, 0.987], *P* = 0.033). The four data points were missing because of a low signal‐to‐noise ratio. (C) In the balance task, the WMH volume increment was strongly positively correlated with the fNIRS data gap in the left orbitofrontal cortex (OFC, r = 0.786, 95% CI [0.182, 0.9859], *P* = 0.028)). The two data points were missing because of a low signal‐to‐noise ratio.

Among these patients, only 10 (4 with svMCI and 6 with aMCI) underwent follow‐up brain MRI and fNIRS after 2 years. The three outliers with excessively severe WMH burden (modified Fazekas grade 3) were not in the follow‐up group. For these 10 patients, we assessed the relationship between WMH volume and fNIRS signal changes at baseline and 2‐year follow‐up for each cognitive and physical task. WMH volume changes showed strong positive correlations with the fNIRS signal changes in the right VLPFC during the SEMT (r = 0.886, 95% CI [0.264, 0.987], *P* = 0.033; Figure 4B) and in the left OFC during the balance tasks (r = 0.786, 95% CI [0.182, 0.959], *P* = 0.028; Figure 4C).

## 4 Discussion

In this study, 27 patients with MCI were classified into the svMCI and aMCI groups based on baseline WMH volume. All patients underwent brain MRI and fNIRS at baseline; among them, 10 underwent follow‐up brain MRI and fNIRS after 2 years. To validate the quantitative WMH cutoff, we also applied the modified Fazekas scale, a widely used visual rating method. This analysis revealed that all svMCI participants were rated as having moderate to severe WMH burden, whereas most aMCI participants were rated as mild. This consistency across methods provides further support for the observed group distinction, though external validation in an independent cohort has not yet been performed. To minimize the potential confounding effects of vascular risk factors, we confirmed that hypertension, diabetes, and APOE4 status did not significantly differ between the svMCI and aMCI groups at baseline. However, subtle influences from subclinical vascular or metabolic dysfunction may still exist.

At baseline, the svMCI group showed a significant decrease in fNIRS signals compared to the aMCI group in the left OFC during the VFT task. After two years, the svMCI group demonstrated further significant decreases in fNIRS signals compared to the aMCI group during the same task, with decreases extending across the bilateral prefrontal cortex. In addition, there is a strong negative correlation between baseline WMH volume and fNIRS signals during the STRC task in the svMCI group. Furthermore, changes in WMH volume from baseline to the 2‐year follow‐up showed strong positive correlations with changes in fNIRS signals during the SEMT and balance tasks. Taken together, these findings highlight distinct patterns of fNIRS activation associated with WMH burden and progression in svMCI, suggesting that fNIRS may serve as a sensitive neuroimaging marker for detecting cerebrovascular changes over time. Our first major finding was that, at baseline, the svMCI group exhibited a significantly lower fNIRS signal in the left OFC during the VFT task compared to the aMCI group, while VFT performance did not differ (Figure 2A). After two years, the decrease in the fNIRS signal in the svMCI group compared to that in the aMCI group extended to the bilateral prefrontal cortex (Figure 2B). The performance on VFT reflects language and frontal function (Piatt et al. 1999), which are associated with activation of the prefrontal cortex (Herrmann et al. 2003). In our study, a significant difference was observed in the left OFC of the prefrontal cortex. It is well established by fNIRS and fMRI studies that the left OFC plays a key role in inhibiting dominant responses and is involved in verbal production, due to its proximity to Broca's area (Yeung 2022).

During the VFT task, the svMCI group exhibited significantly decreased fNIRS signals compared to the aMCI group. We interpret this decrease as potentially reflecting inefficient compensatory mechanisms in patients with svMCI, in contrast to the compensatory activation observed in patients with aMCI. Supporting this interpretation, previous fNIRS and ASL‐MRI studies have demonstrated compensatory activation characterized by increased CBF in the prefrontal cortex of aMCI patients (Duan et al. 2020; Yap et al. 2017). Additionally, a previous pilot fNIRS study on patients with CSVD reported neurovascular uncoupling during cognitive tasks, suggesting that impaired neurovascular regulation may contribute to the limited compensatory response observed in svMCI (Wen and Xu 2023). Conversely, during the SEMT and balance tasks, the svMCI group exhibited significantly increased fNIRS signals compared to the aMCI group. However, these findings were inconsistent between baseline and the 2‐year follow‐up assessments. Specifically, during the SEMT task, increased fNIRS activation in the svMCI group was evident only at baseline, whereas during the balance task, significant increases were noted exclusively at the 2‐year follow‐up. Given these temporal inconsistencies, we acknowledge that the observed fNIRS signals may have been influenced by measurement variability or noise.

Interestingly, despite the distinct fNIRS patterns, no significant differences were observed in neuropsychological test performance between svMCI and aMCI. Although neuropsychological tests are sensitive, they have inherent limitations. For instance, whether a participant solves a problem effortlessly or with great effort, the final score remains the same, potentially failing to capture differences in cognitive processing. In our study, the svMCI group exhibited longer response times and lower accuracy on several cognitive tasks during fNIRS signal acquisition compared to the aMCI group, despite no significant differences in cognitive profiles assessed by conventional neuropsychological tests. In SVCI, it is well established that memory function is initially preserved, while deficits in information processing speed—an essential component of cognitive processing—are among the most commonly affected domains (Lu et al. 2024). The observed fNIRS differences suggest that fNIRS may capture cognitive effort or compensatory response during processing, which neuropsychological test scores alone may not fully capture.

Our second major finding was a strong negative correlation between baseline WMH volume and fNIRS signal in the svMCI group during the STRC task (Figure 3). Several previous studies have revealed that an increase in WMH burden was associated with a decrease in resting‐state CBF using ASL‐MRI in the elderly (Bangen et al. 2021), using SPECT in MCI patients (Ishibashi et al. 2018), and CT perfusion imaging in AD patients (Li et al. 2019). Furthermore, it has been established that lower cerebrovascular reactivity (CVR) in response to hypercapnia is related to a more severe WMH burden in CSVD patients (Blair et al. 2020; Sleight et al. 2023) and the elderly (Sam et al. 2016a; Sam et al. 2016b; Sam et al. 2016c; Sam et al. 2016d). Impaired CVR contributes to neurovascular dysfunction, reducing adaptive perfusion and increasing susceptibility to ischemic injury in CSVD (Sleight et al. 2023), which may lead to reduced fNIRS responses in svMCI. The pathophysiology of CSVD is associated with endothelial dysfunction (Quick et al. 2021), blood‐brain barrier (BBB) dysfunction (Bridges et al. 2014), arterial stiffness (Okada et al. 2014), and neuronal and glial cell degeneration, all of which may contribute to impaired hemodynamic responses. Among these factors, recent evidence suggests that BBB dysfunction plays a key role in neurovascular uncoupling in small vessel disease (Hosoki and Sachdev 2024).

Interestingly, three outliers with excessively severe WMH burden (modified Fazekas grade 3) did not demonstrate an association with fNIRS signals, suggesting a deviation from normal CVR regulation (Supplementary Figure 3). Consistent with our findings, previous studies have shown that severe WMH burden may exhibit alterations in CBF distinct from those observed in mild‐to‐moderate WMH cases (Huang et al. 2021). Overall, our results highlight the ability of the STRC task to reveal the association between WMH burden and fNIRS activation in svMCI patients. To our knowledge, this is the first study to demonstrate that cerebrovascular hemodynamics in response to cognitive tasks are related to greater WMH burden in svMCI patients, despite the limitation of a small sample size.

Notably, a negative correlation between fNIRS signals and WMH volume was observed during STRC tasks; however, this correlation was not evident during STRI tasks. Participants tend to exert more effort during the STRI task performed after the STRC task (Blais et al. 2014). This additional effort could potentially provoke higher neural activity, which may obscure the correlation observed during the STRC task. Consequently, this phenomenon could explain why a negative correlation between fNIRS signals and WMH volume was observed during STRC tasks but not during STRI tasks.

Our third major finding was a strong positive correlation between WMH volume progression from baseline to the 2‐year follow‐up and fNIRS signal changes observed during the SEMT and balance tasks. During the SEMT task, a significant association was observed between the WMH volume progression and fNIRS signal changes in the right VLPFC. The SEMT is a specialized task designed to evaluate verbal, visual, episodic, and associative memory abilities (Kim et al. 2018). Previous studies have highlighted the role of the right VLPFC in connections with temporal lobe regions specializing in face and object recognition (Bunge et al. 2004; Lee et al. 2013), as well as its involvement in processes related to subsequent episodic memory (Weintraub‐Brevda and Chua 2019), which are involved in the SEMT task. During the balance task, a significant association was observed between WMH burden progression and fNIRS signal changes in the left OFC. Previous studies have noted OFC activation during exercise tasks using fNIRS and fMRI (Claus et al. 2023; Miyashiro et al. 2021). Consistent with our results, studies investigating CBF using ASL‐MRI and phase‐contrast MRI found that low CBF predicts WMH progression (Bernbaum et al. 2015; Promjunyakul et al. 2018; Staffaroni et al. 2019; ten Dam et al. 2007; Van Der Veen et al. 2015). Another study investigated CBF using resting (^15^O‐)H_2_O PET in patients with WMH and found that the patterns of regional CBF were different between the WMH progression group and the non‐progression group over the 8‐year follow‐up (Kraut et al. 2008). On the other hand, a study focusing specifically on a healthy elder population (n = 252) found no associations between baseline CBF and WMH growth (Nylander et al. 2018). Taken together, the relationship between resting CBF and WMH remains unclear. However, our findings suggest that WMH progression is associated with fNIRS signal changes and that the SEMT and balance tasks are effective in revealing this association in patients with MCI over a 2‐year period, although these correlational findings should be interpreted with caution due to the small sample size.

This study has several limitations. First, because of the small sample size (n = 27; 10 at follow‐up), which is appropriate for a pilot study (Cocks and Torgerson 2013; Whitehead et al. 2016), the influence of noise cannot be ruled out and may increase the risk of overinterpreting the findings, particularly in correlational analyses. In addition, the absence of a healthy control group limits our ability to interpret whether observed fNIRS patterns represent deviations from normal aging or distinct pathological processes. Second, the fNIRS device used in this study measures signals exclusively in the prefrontal cortex. While this may limit whole‐brain interpretation, it remains relevant given the predominant distribution of WMH in the prefrontal subcortex. Finally, as this study was conducted in an Asian population, where CSVD is more prevalent (Hilal et al. 2017), genetic, cultural, and environmental factors may have influenced cerebrovascular pathology and fNIRS responses. Therefore, the generalizability of our finding to other ethnic groups or regions remains uncertain. Further studies with large cohorts involving more diverse populations are needed to validate these results.

## 5 Conclusion

In conclusion, our study highlights the consistent effectiveness of the VFT task in differentiating fNIRS activation between svMCI and aMCI groups at both baseline and 2‐year follow‐up. Furthermore, the STRC task revealed a significant association between baseline WMH burden and fNIRS activation in svMCI patients. Finally, the SEMT and balance tasks demonstrated their utility in linking WMH progression to changes in fNIRS signals over the 2‐year period. These findings underscore the potential of fNIRS signals as a biomarker for detecting hemodynamic changes associated with WMH burden and predicting WMH progression. Future research should pursue multicenter validation of fNIRS‐based biomarkers, incorporate CVR and white matter microstructure (e.g., DTI), and leverage machine learning to analyze multimodal data for improved diagnostic accuracy in SVCI.

## Author Contributions

**Qi Wang**: data curation, formal analysis, methodology, writing–original draft, funding acquisition, visualization. **Byoung‐Soo Shin**: resources, writing–review and editing. **Sun‐Young Oh**: resources, writing–review and editing. **Seungbae Hwang**: resources, writing–review and editing. **Ko Woon Kim**: conceptualization, methodology, formal analysis, resources, writing–review and editing, funding acquisition, supervision.

## Ethics Statement

This study was approved by the Institutional Review Board of the Jeonbuk National University Hospital (2020‐12‐023).

## Consent

All the participants provided written informed consent after the study objectives were explained.

## Conflicts of Interest

The authors declare no conflicts of interest.

### Peer Review

The peer review history for this article is available at https://publons.com/publon/10.1002/brb3.70598

## Supporting information



Supporting Information

## Data Availability

The data that support the findings of this study are available from the corresponding author upon reasonable request.
