# Supplementary material for: Functional Near‐Infrared Spectroscopy Signal as a Potential Biomarker for White Matter Hyperintensity Progression in Patients With Subcortical Vascular Cognitive Impairment: A Pilot Study
Source: Brain Behav. 2025 May 30;15(6):e70598. doi: 10.1002/brb3.70598 (PMC12123445; doi:10.1002/brb3.70598)
Supplement: Supplementary file 1 — Supporting Information [file BRB3-15-e70598-s001.docx]

**Supplementary** **Information**

**S1 Detailed description of** **cognitive and physical tasks**

**S1****.1 Cognitive Tasks**

S1.1.1 VFT

The performance in the VFT assessed various aspects of language and frontal function, including vocabulary quantity, maintenance or updating, selection, manipulation, and lexical access speed(Piatt et al., 1999). The entire procedure consisted of three blocks, each comprising a 30-second initial rest period followed by a 60-second VFT (with 30 seconds allocated for pre-task and 30 seconds for the actual task). During the pre-task, participants were asked to read out the five Korean vowels, "a", "ae", "i", "o", and "u". In the actual task, participants were instructed to produce as many Korean words as they could that began with a specific randomly presented vowel among the five vowels mentioned above.

S1.1.2 DST

This task assesses the participant's verbal short-term and working memory abilities (Baddeley, 1992; Risberg and Ingvar, 1973; Wechsler, 1940). The task involves the participant repeating a series of digits in reverse order, as presented on the screen. Initially, a sequence of 3 digits is provided, followed by a sequence of 4 digits. The participant inputs their digit sequence into the tablet computer screen.

S1.1.3 K-CWST

K-CWST is comprised of the congruent condition (STRC) and the incongruent condition (STRI) which are indicative of frontal-executive function(Amato et al., 2006; Jensen and Rohwer, 1966). In this study, a modified version of the K-CWST was employed, where participants were instructed to respond by touching the tablet computer screens. In the STRC, colored words were presented in a consistent color, and participants were required to touch the word that matched the color shown. Conversely, in the STRI, color-words were presented in an inconsistent color (e.g., the word "red" displayed in green ink), and participants were instructed to touch the color name that corresponded to the ink color rather than the word itself.

S1.1.4 SEMT

The SEMT was specifically developed to assess verbal, visual, episodic, and associative memory abilities (Kim et al., 2018). Before initiating the task, participants were to memorize the conversation between the performers to the best of their ability. Subsequently, participants were presented with a 7-minute video clip and were tasked with answering questions about specific details from the video on the tablet computer. Each question provided six response options, and participants were required to select and input their answers into the tablet computer.

**S1.2 Physical Tasks**

S1.2.1 Balance task

Specifically, the "balance task" consisted of the following sequence: 30 seconds of rest in a standing position, followed by a 20-second task where participants maintained balance on one leg with their eyes open and arms extended, then another 32 seconds of rest in a standing position, followed by a 20-second task where participants maintained balance on one leg with their eyes closed and arms extended, and finally, another 32 seconds of rest in a standing position.

S1.2.2 Squat task

For the "squat task", the sequence was as follows: 60 seconds of rest in a standing position, followed by a 60-second task where participants maintained a squatting position, and finally, 63 seconds of rest in a standing position.

| Abbreviation | Full Name | Task Description |
| --- | --- | --- |
| VFT | Verbal Fluency Test | Participants generate as many words as possible within a category in 60 seconds, reflecting language and frontal function. |
| DST | Digit Span Backward Task | Participants recall sequences of numbers in reverse order to assess working memory. |
| STRC | Stroop Test – Congruent Condition | Participants read color words printed in matching ink colors (e.g., “red” in red), measuring attention. |
| STRI | Stroop Test – Incongruent Condition | Participants name the ink color of color words that mismatch the text (e.g., “red” in blue), assessing inhibitory control. |
| SEMT | Social Event Memory Test | Participants view a short video and answer questions assessing associative and episodic memory. |
| Balance | Balance Task | Participants stood on one leg with arms extended, eyes open and closed, assessing motor control. |
| Squat | Squat Task | Participants alternated between standing and squatting for 60 seconds to measure physical exertion-related brain activity. |

**Supplemen****tary Table 1. Summary of Cognitive and Physical Tasks Abbreviations and Descriptions.**


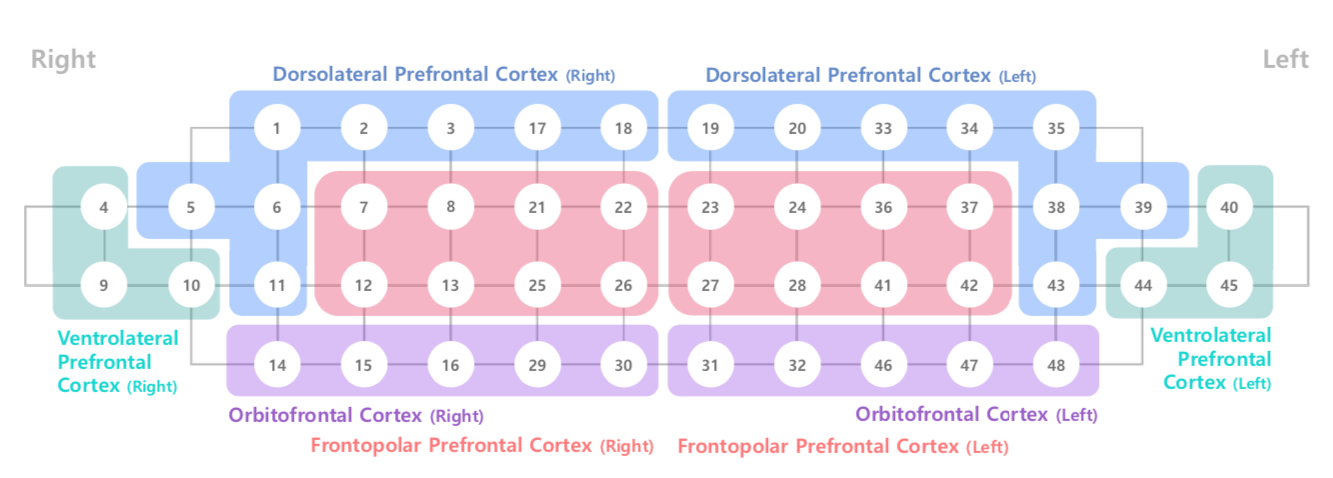
 **Supplementary Figure 1.** **Schematic diagram illustrating the designated channels of the fNIRS device corresponding to each Brodmann region.**


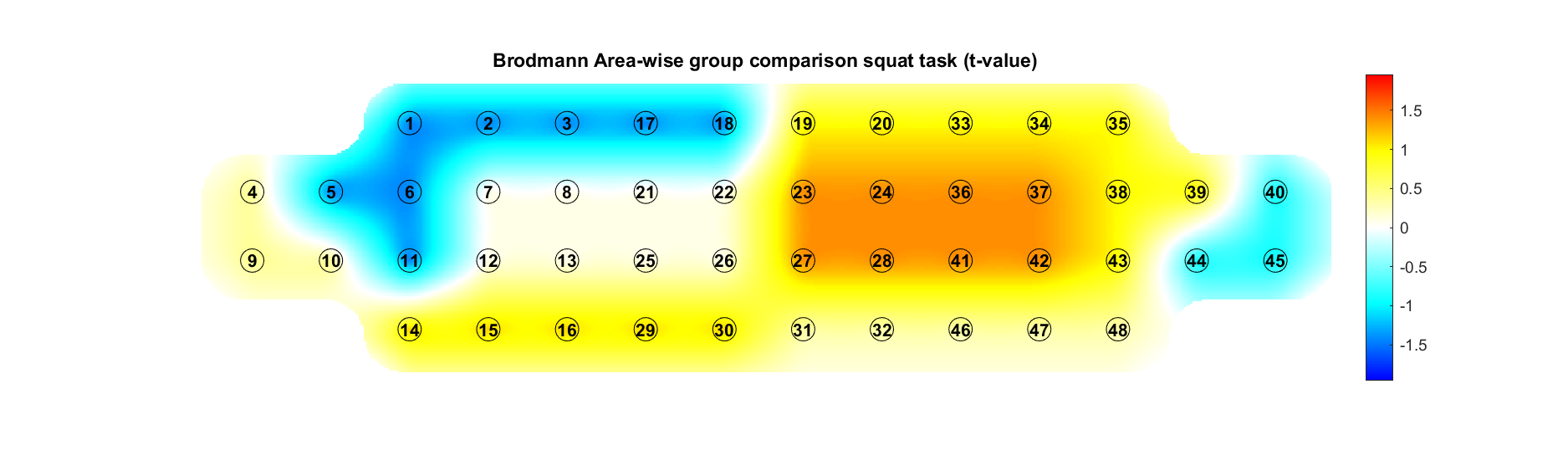

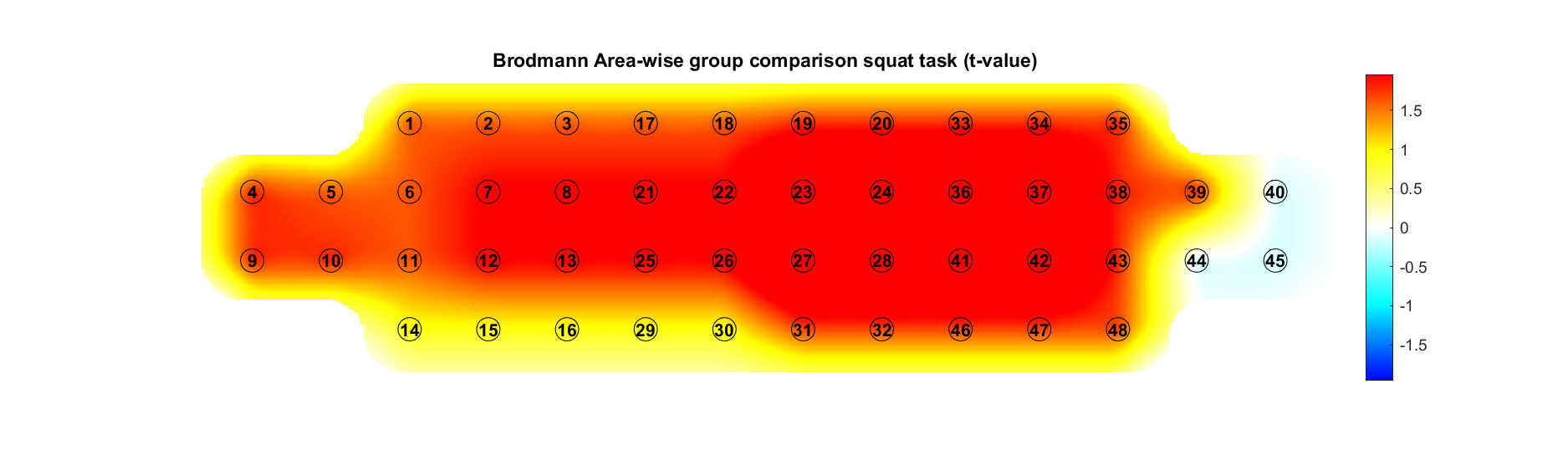

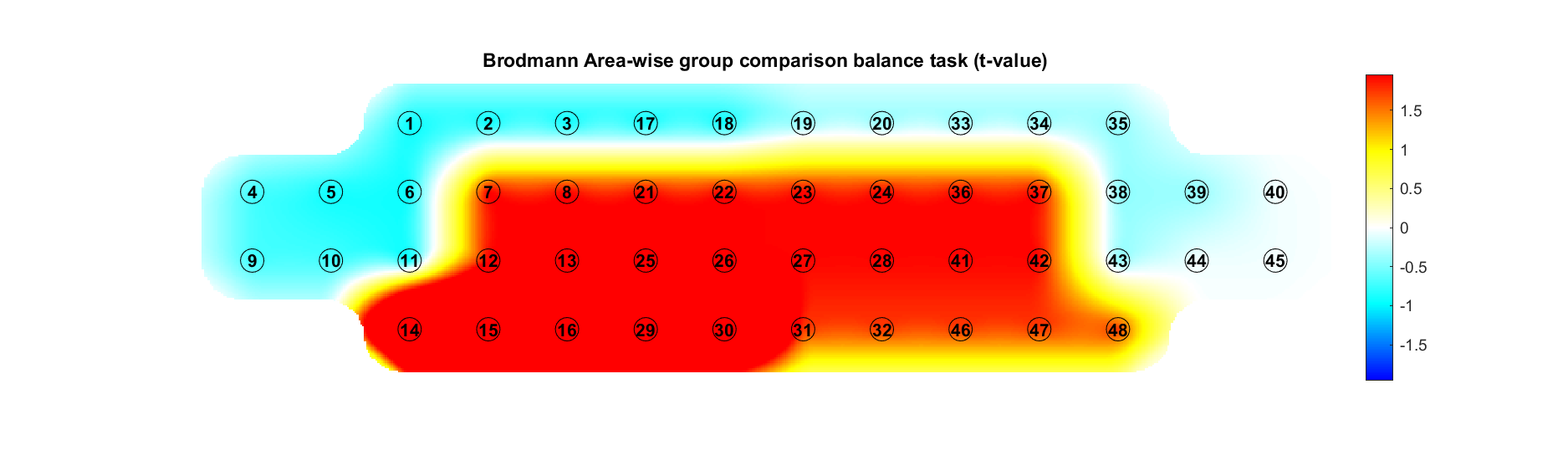

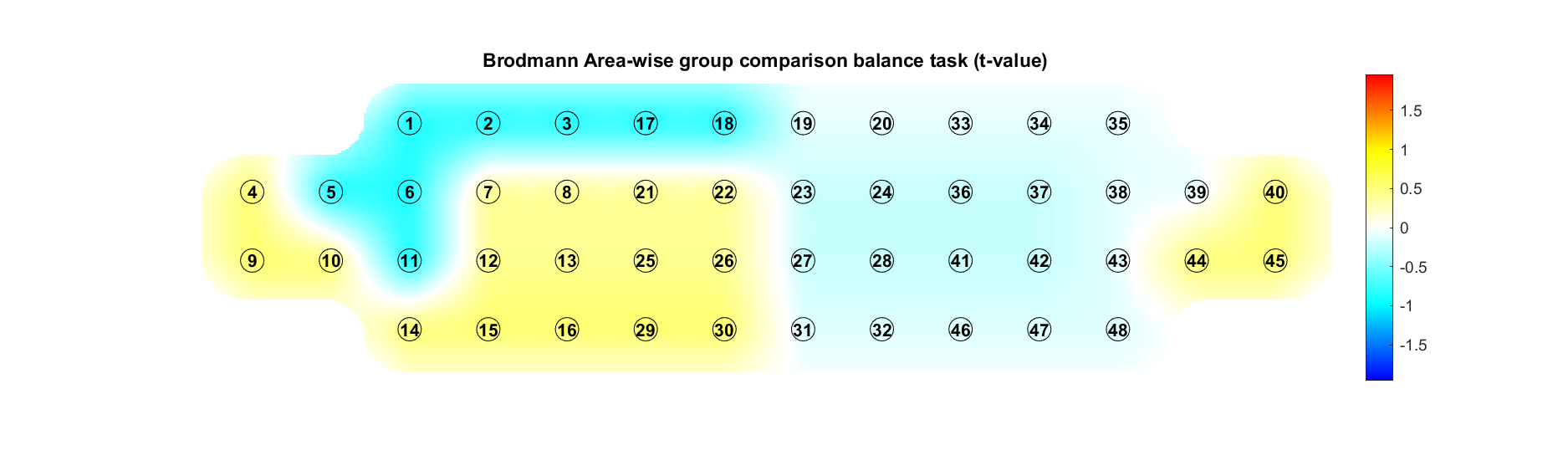

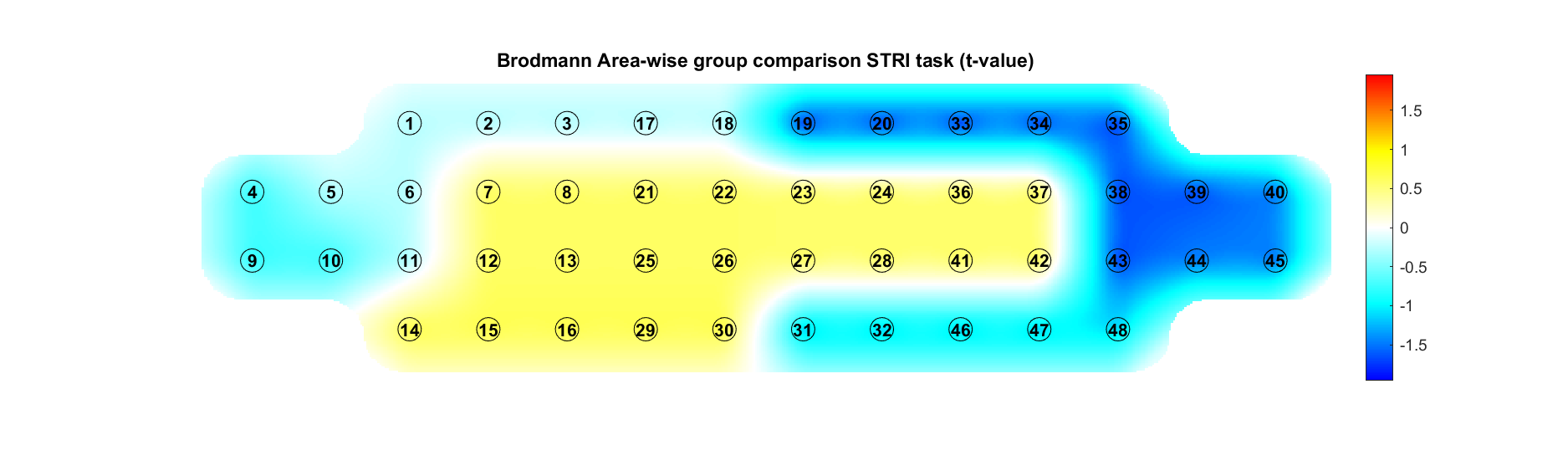

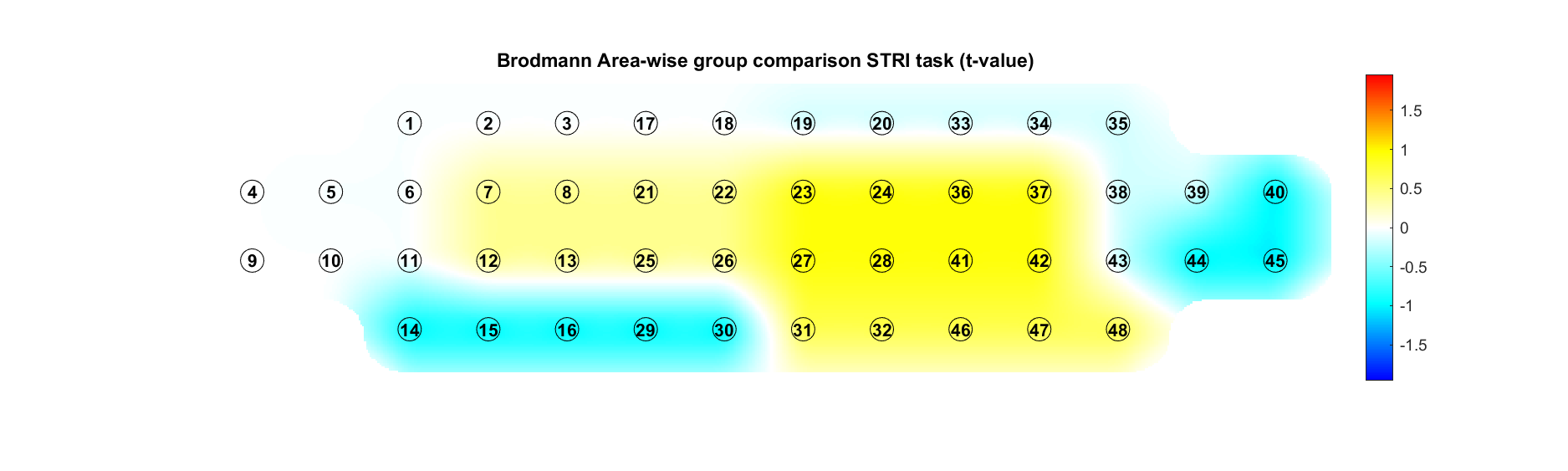

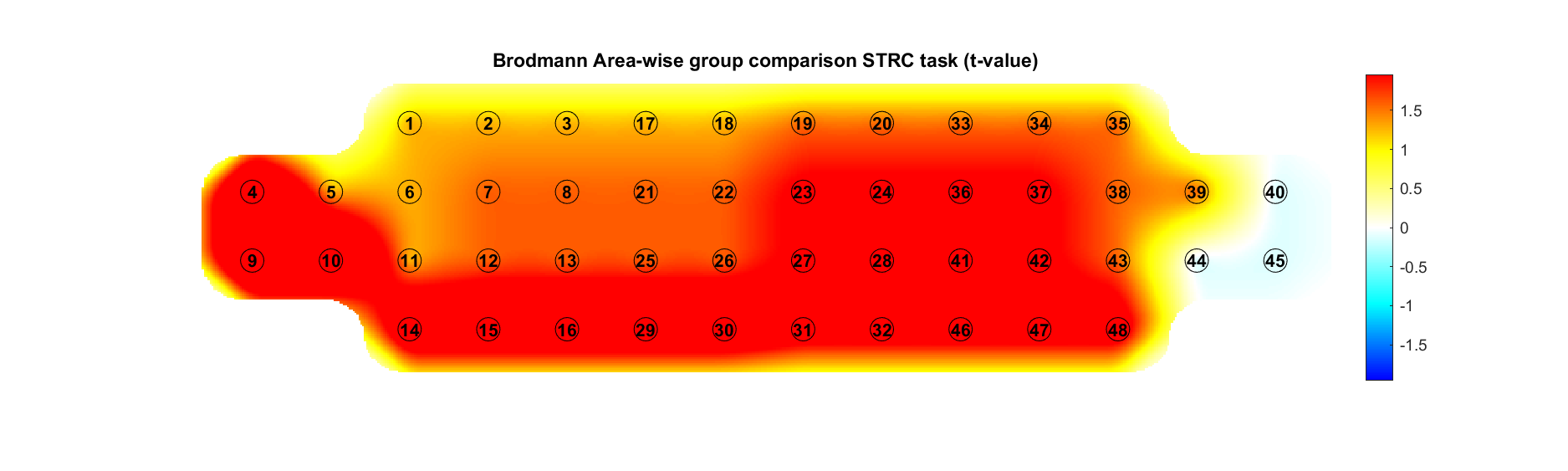

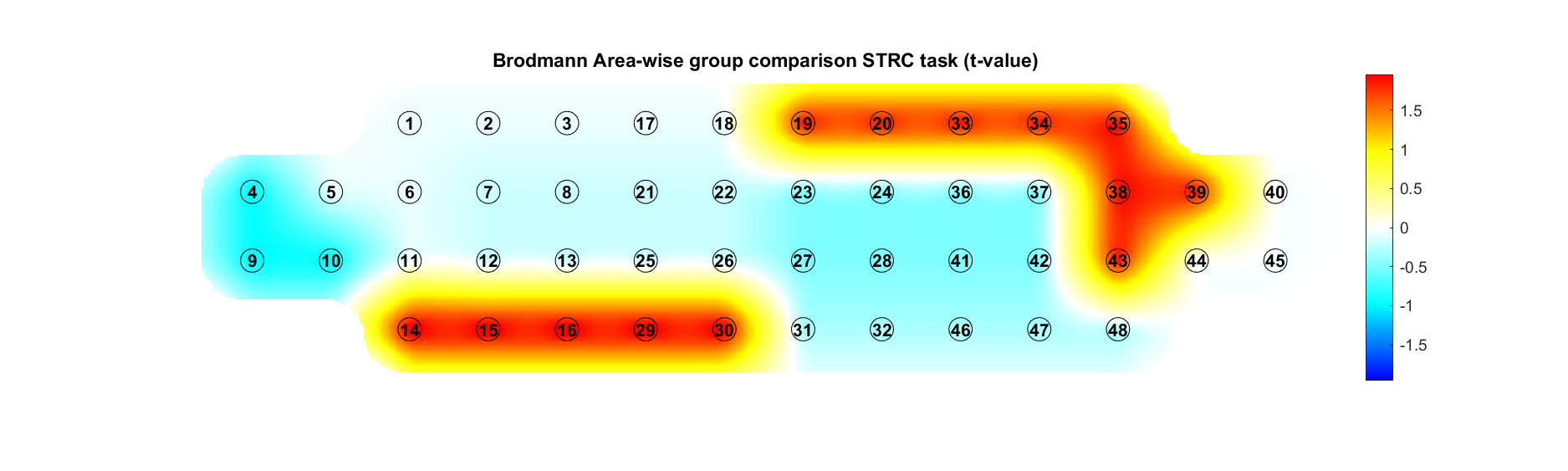

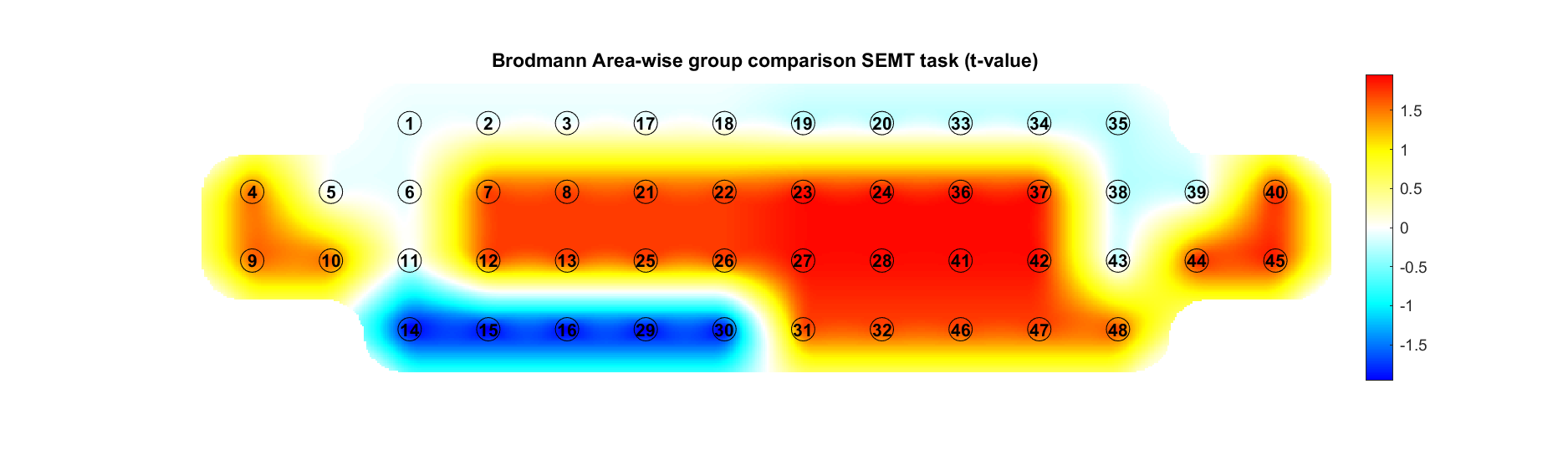

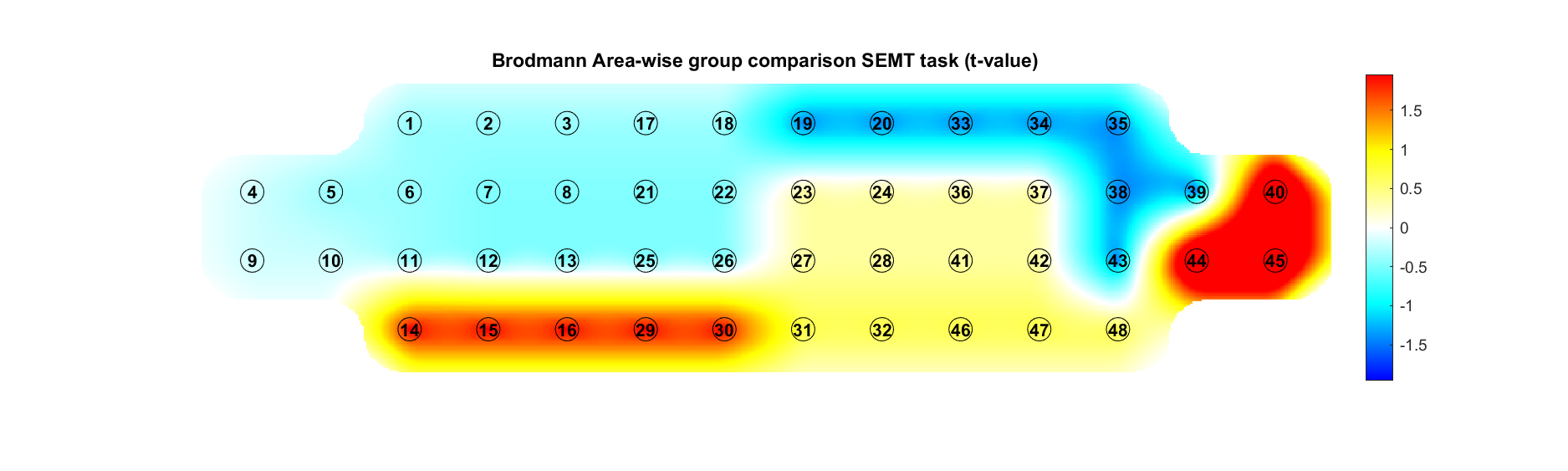

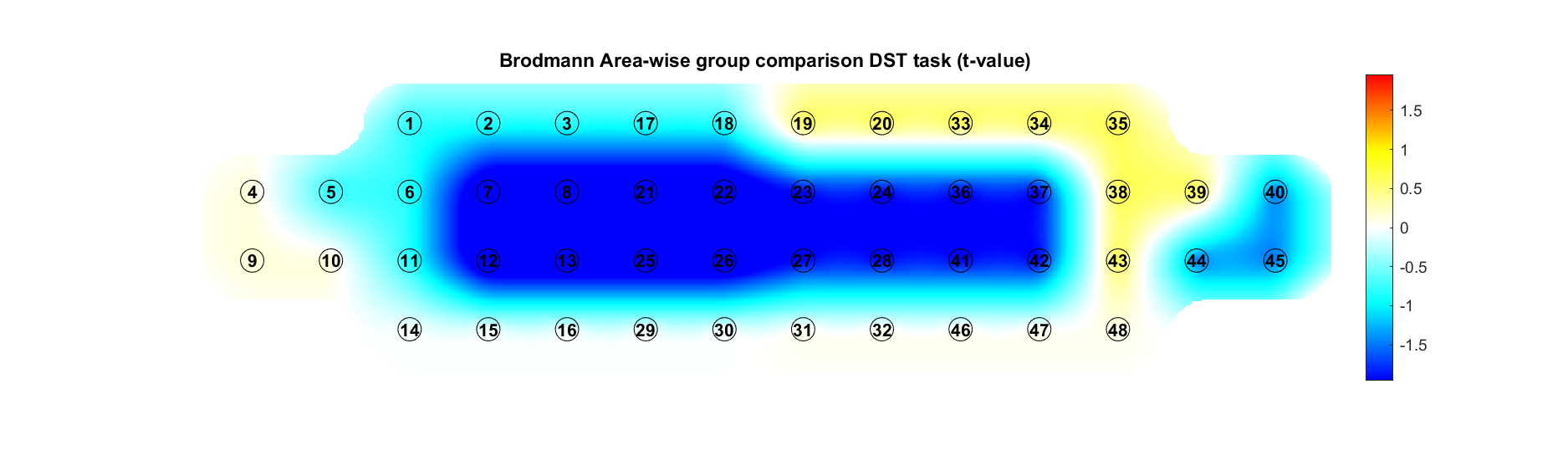

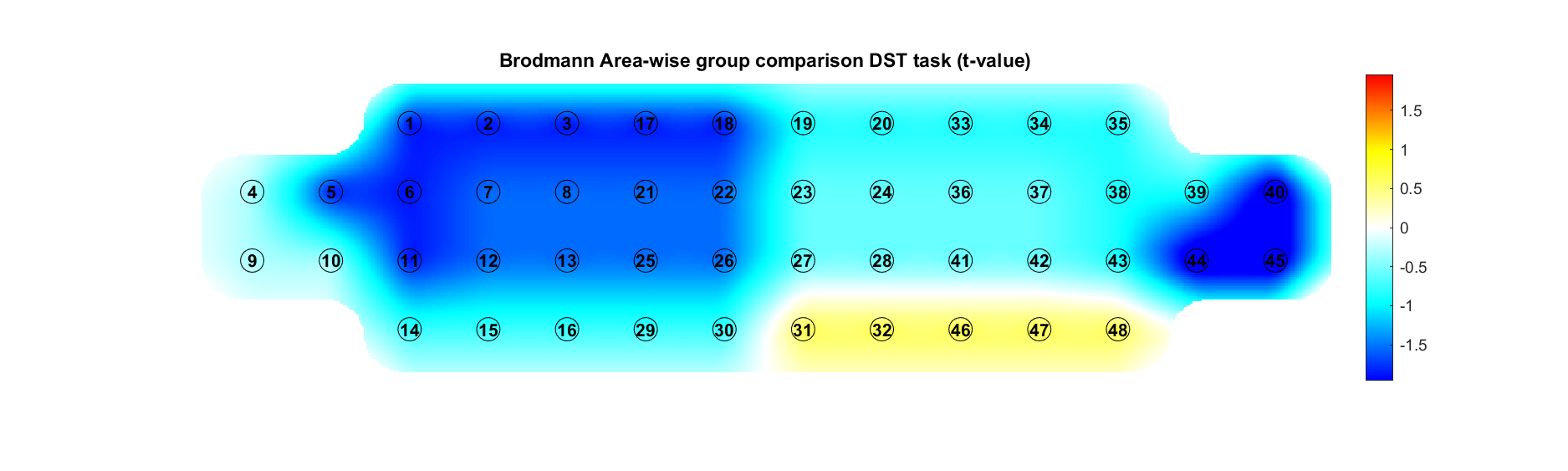


A-1

A-2

B-1

B-2

C-1

C-2

D-2

D-1

E-1

E-2

F-1

F-2

**Supplementary Figure 2. Comparison of functional near-infrared spectroscopy (fNIRS) data between subcortical vascular mild cognitive impairment (svMCI) group and amnestic mild cognitive impairment (aMCI) group of baseline and the 2-year follow-up.** The red color indicates increased fNIRS data and the blue color indicates decreased fNIRS data in the svMCI group. There was no significant difference between svMCI and aMCI group in (A-1) baseline and (A-2) 2-year follow-up of the Verbal Fluency test. (B-1) In the SEMT task, the svMCI group exhibited a significantly increased fNIRS signal compared to the aMCI group in the left VLPFC (T = 3.526, Cohen’s d = 1.962, 95% CI [3.290, 0.635], *P* = 0.03) at the baseline, however, no significant difference was found in (B-2) 2-year follow-up. As for the (C-1) baseline and (C-2) 2-year follow-up of the Korean-color word stroop test congruent condition, (D-1) baseline and (D-2) 2-year follow-up of the Korean-color word stroop test incongruent condition, no significance was observed. In the balance task, (E-1) the fNIRS data did not significantly during the baseline visit, but (E-2) at the 2-year follow-up, svMCI patients showed a significant increase of fNIRS data compared to that of aMCI patients in the right orbitofrontal cortex (T = 5.820, Cohen’s d = 5.432, 95% CI [9.394, 1.471], *P* = 0.022). And in the squat task, (F-1) baseline and (F-2) 2-year follow-up showed no significant difference.

**
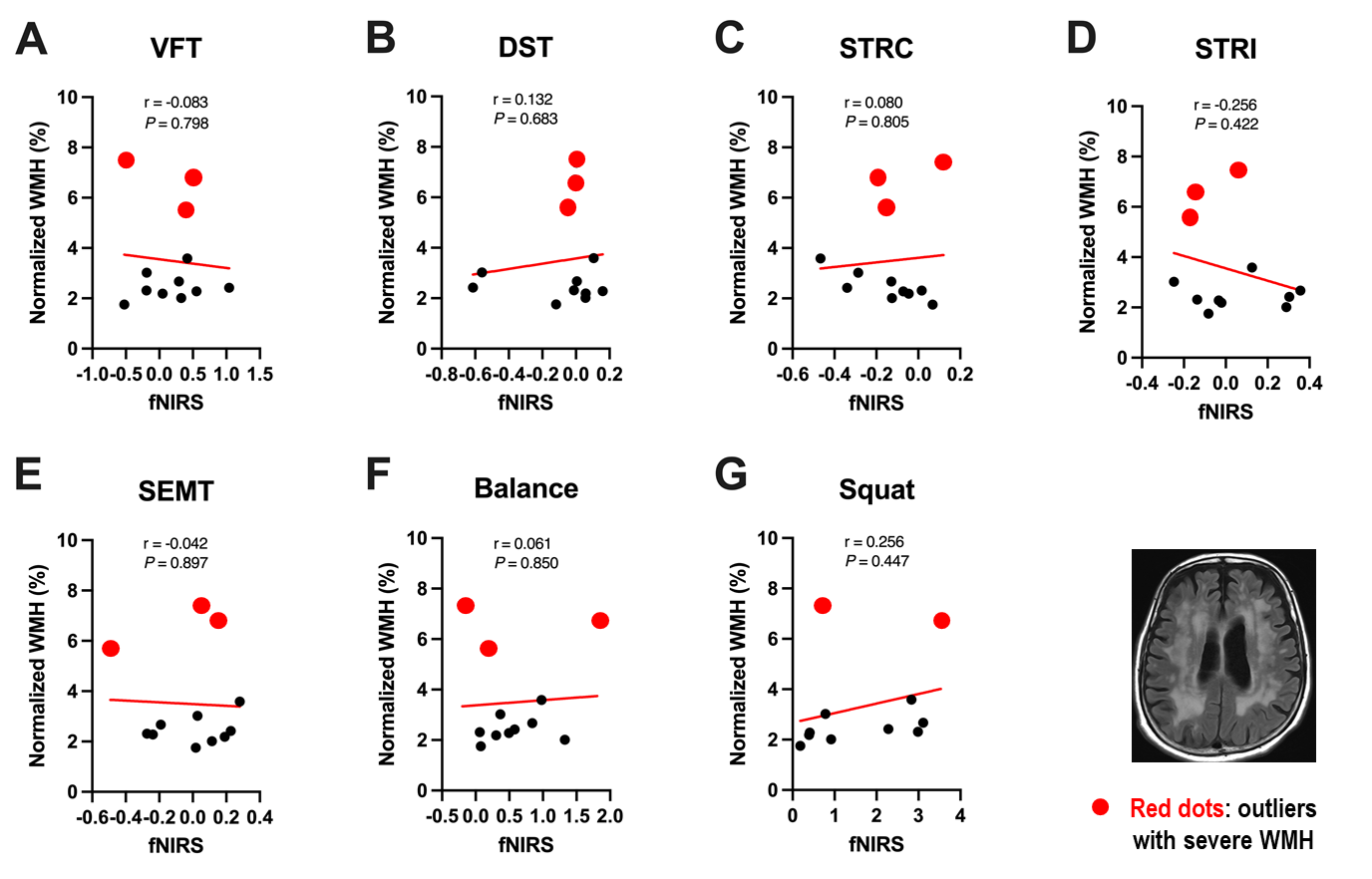
**

**Supplementary Figure 3. Correlation between baseline functional near-infrared spectroscopy (fNIRS) data and white matter hyperintensity (WMH) volume during cognitive and physical tasks in the svMCI group, including three outliers.**

The correlation between baseline WMH volume and fNIRS signals during the STRC task was not significant when outliers were included (r = 0.080, *P* = 0.805, Figure C).

**Supplementary references**

Amato MP, et al. The Rao's Brief Repeatable Battery and Stroop Test: normative values with age, education and gender corrections in an Italian population. Mult. Scler. 2006; 12(6): 787-93. 10.1177/1352458506070933

Baddeley A. Working Memory. Science 1992; 255(5044): 556-9. 10.1126/science.1736359

Jensen AR, Rohwer WD, Jr. The Stroop color-word test: a review. Acta Psychol. 1966; 25(1): 36-93. 10.1016/0001-6918(66)90004-7

Kim KW, et al. Social Event Memory Test (SEMT): A Video-based Memory Test for Predicting Amyloid Positivity for Alzheimer's Disease. Sci. Rep. 2018; 8(1): 10421. 10.1038/s41598-018-28768-1

Piatt AL, et al. Action (verb naming) fluency as an executive function measure: convergent and divergent evidence of validity. Neuropsychologia 1999; 37(13): 1499-503. 10.1016/s0028-3932(99)00066-4

Risberg J, Ingvar DH. Patterns of Activation in the Grey Matter of the Dominant Hemisphere during Memorizing and Reasoning: A Study of Regional Cerebral Blood Flow Changes during Psychlogical Testing in a Group of Neurologically Normal Patients. Brain 1973; 96(4): 737-56.

Wechsler D. The measurement of adult intelligence. J. Nerv. Ment. Dis. 1940; 91(4): 548. 10.1097/00005053-194004000-00075
